# Female Reproductive Health in SARS-CoV-2 Pandemic Era

**DOI:** 10.22074/IJFS.2021.534956.1164

**Published:** 2021-10-16

**Authors:** Reihaneh Nateghi, Shahriar Ghashghaei, Bahare Shokoohian, Maryam Hezavehei, Mahkameh Abbaszadeh, Bita Ebrahimi, Abolhossein Shahverdi, Mehri Mashayekhi, Anastasia Shpichka, Peter Timashev, Mohammad Hossein Nasr-Esfahani, Massoud Vosough

**Affiliations:** 1 Department of Embryology, Reproductive Biomedicine Research Center, Royan Institute for Reproductive Biomedicine, ACECR, Tehran, Iran; 2 Department of Regenerative Medicine, Cell Science Research Center, Royan Institute for Stem Cell Biology and Technology, ACECR, Tehran, Iran; 3 Reproductive Biomedicine Research Center, Royan Institute for Reproductive Biomedicine, ACECR, Iran, Tehran; 4 Institute for Regenerative Medicine, Sechenov University, Moscow, Russia; 5 Department of Reproductive Biotechnology, Reproductive Biomedicine Research Center, Royan Institute for Biotechnology, ACECR, Isfahan, Iran

**Keywords:** COVID-19 Pandemic, Female Infertility, Female Reproductive Health, Fetal Development, SARS-CoV-2

## Abstract

Severe acute respiratory syndrome coronavirus 2 (SARS-CoV-2) pandemic struck global health systems with over- growing demands in many fields of health care; yet, reproductive care, particularly pregnancy care remains a special focus of interest. Pregnancy is a major physiologic change that alters temporarily normal function of many organs, and specifically the immune system. Therefore, pregnant women are more susceptible to respiratory pathogens compared to the others. The current pandemic may have serious consequences on pregnancy whether directly or indirectly. In the present review, direct and indirect possible adverse effects of SARS-CoV-2 infection on female reproductive system by focusing on pregnancy and delivery has been discussed in details. In addition, the pregnancy consequences and whether maternal infection can affect infants were deliberated. The adverse impact of luck down and related psycho- logical complications and obesity on pregnant women were discussed as well. Finally, the effects of SARS-CoV-2 vaccination on maternal health and pregnancy outcome was analyzed.

## Introduction

Health systems are challenged by overwhelming requests created by SARS-CoV-2 pandemic. Yet, reproductive medicine including pregnancy care remains as an essential part of health services requiring special attention (1). Pregnancy makes changes on the immunity status and might make pregnant women more susceptible to respiratory pathogens and pneumonia (2). Pregnancy results in physiological adaptions such as airway edema, diaphragmatic elevation, more oxygen consumption, and pregnancy-related immune alterations (3). Moreover, swelling of upper respiratory tract because of high levels of estrogen and progesterone in addition to limited lung expansion capacity lead to the vulnerability of the pregnant woman to the respiratory pathogens (2, 3). Different processes in female reproductive system, including folliculogenesis, steroidogenesis, oocyte maturation are regulated by renin-angiotension aldosterone system (RAAS) that comprises the classic components of angiotensin converting enzyme (ACE), angiotensin 2 (Ang2) and angiotensin II type 1 receptor (AT1R) axis along with new discovered components i.e. Ang [1-7] and Mas. Angiotensin-converting enzyme 2 (ACE2) and transmembrane serine protease 2 (TMPRSS2) play the key role as entry receptors for SARS-CoV-2. Expression of ACE2 and TMPRSS2 was not only detected in epithelial cells and stromal cells of endometrium throughout the whole menstrual cycle (4); but also, the presence of these receptors were identified during first, second and third trimester of pregnancy (5). Moreover, during embryogenesis, ACE2 was identified in inner cell mass and trophoblast while TMPRSS2 was only seen in trophoblast. On contrast, none had significant expression in oocytes and cleavage embryos. Therefore, at each stage, certain cells are susceptible to infection by SARS-CoV-2. This paper focused on direct and indirect possible adverse effects of SARS-CoV-2 infection on the female reproductive health systems, the pregnancy consequences and whether maternal infection affects infants. Finally, the effects of SARS-CoV-2 vaccination on maternal health and pregnancy outcome was discussed.

### SARS-CoV-2 and female reproductive health system

Studies suggested that SARS-CoV-2 might cause dysfunction in the female reproductive system, directly or indirectly. The direct adverse effects are related to cytopathic impact of virus colonization and the indirect effects are associated with exacerbation caused by RAAS, inflammatory reactions, psychological disorders, and obesity.

### Tissue distribution of ACE2 in the female reproductive system

The expression of ACE2 in human ovaries and endometrium has been reported. Throughout menstrual cycle, expression of ACE2 in endometrium changes based on the phase of cycle. In proliferative phase, expression of ACE2 is predominant in epithelial cells while in secretory phase, significant expression of this receptor is evident in both epithelial and stromal cells (6).

Data regarding the expression of ACE2 in oocytes and embryos are controversial. Previous publications indicated that high levels of ACE2 is expressed in the germ cells and early embryos (7) while some recent data reported the opposite. Recently, Stanley and colleagues revealed that co-expression of ACE2 and TMPRSS2 increased during oocyte maturity, therefore, primordial follicles have less susceptibility to the infection compared to the more matured follicles. Regardless, the study suggests that possibility of transient effects is low. In addition, ACE2 expression in human cumulus cells was reported, though TMPRSS2 expression was very low in the cumulus cells. Therefore, it seems that there is a low risk for infection in these type of cells (8). In contrast to the previous findings, Reis et al. (9) found that there is a slight possibility of presence of ACE2 and TMPRSS2 in oocytes. Furthermore, ACE2 was detected in follicular fluid (FF).

Although there were ACE2 receptors in the female reproductive tract, but there is no strong evidence for the virus colonization through ACE2 receptors in the female reproductive system so far.

### Renin-angiotensin aldosterone system in COVID-19

There is a substantial correlation between RAAS components and gonadotropins; meaning that gonadotropins can increase RAAS components’ expression (9) and vice versa (10-12) in addition that both can influence function of ovary (11-13).

High levels of gonadotropins’ induces the expression of Ang (II) in FF (9). ACE2 uses Ang II as its key substrate to produce angiotensin [1-7], exerting vasodilatory activity via the mas receptor (MasR). Ang [1-7] and MasR, in the theca-interstitial cells, could raise the level of ovarian steroidogenesis and regulate the ovary physiologic functions such as follicular development, steroidogenesis, oocyte maturation, ovulation (10). Recently, the ability of ACE2/Ang [1-7]/MasR axis has been proved in enhancement of meiotic resumption and it is well-known that meiotic resumption can be adjusted by luteinizing hormone (12). In addition, regulation of ACE2 expression by gonadotropins, and its contribution in follicular development have been already mentioned (13). Reis and colleagues showed presence of ACE2 and active Ang [1-7]-MasR-ACE2 axis in the human ovarian follicles (9). The gonadotropin-dependent expression of ACE2 in human ovaries has widely covered in the literature, although ACE2 receptors in male reproductive system were more notable than female reproductive system (11, 14).

**Fig 1:**
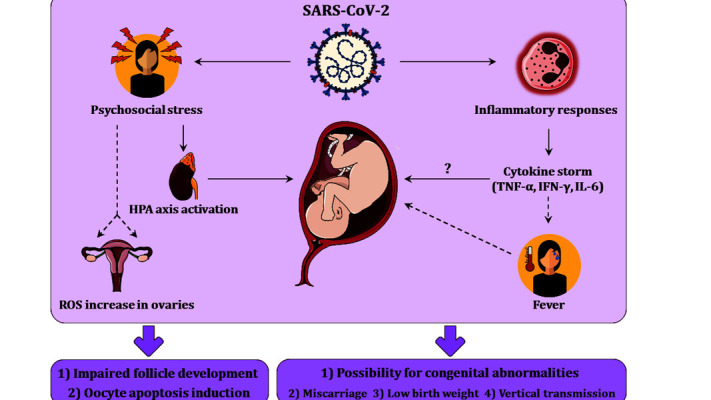
These figure represents different etiological factors affecting female reproductive health system. Inflammatory reactions and psychosocial stress can cause many complications in pregnant mothers.

Due to correlation between female gonadotropins and ACE2 expression- as a part of RAAS system and key entry point for the SARS-CoV-2 -, there is a reasonable possibility of infection exacerbation in female reproductive system. Figure 1 illustrates different etiological pathways in pathogenesis of COVID-19 related female fertility complications.

### SARS-CoV-2 severe inflammatory response and female reproduction

Cytokine-storm is another serious consequence of SARS-CoV-2 infection. The plasma concentrations of different interleukins (IL) and tumor necrosis factor α (TNF-α) raised during SARS-CoV-2 infection which could lead to morbidity or even mortality due to multiple organ failure (15). The toxic effect of TNF on developmental competency was already shown. It was suggested that increased level of TNF-α in the maternal blood might be noxious for early embryo growth (16).

Other study reported that patients with SARS-CoV-2 had higher levels of inflammatory cytokines [TNF-α, interferon-γ (IFN-γ), IL-2, and IL-6] than control individuals (16-18). High levels of IL-6 were associated with the clinical intensity of SARS-CoV-2; thus, IL-6 level could be used as a biomarker in acute phase to determine the severity of infection (19), an independent predictor of mortality (20) and a hallmark for efficacy of possible treatments (21, 22).

### SARS-CoV-2 and psychological factors in female reproduction

Previous studies have shown that viral diseases such as severe acute respiratory syndrome (SARS), Middle East respiratory syndrome (MERS), and H1N1 could initiate serious panic in societies like depression, anxiety, fear, and post-traumatic stress disorder (23, 24). Recent study showed that SARS-CoV-2 pandemic not only causes medical concerns, but also initiates different psychological complications. Frequency of anxiety, stress, and depression were reported to be around 31.9%, 29.6%, and 33.7% respectively in this pandemic (25).

Association between stress and reproductive function impairment in infertile women is acknowledged (26). This correlation could be identified by activating the hypothalamic-pituitary-adrenal (HPA) axis, body-stress response and dysregulation in hormones (27). Stress could increase reactive oxygen species (ROS) and oxidative stress in the ovaries, which lead to restricted development of follicles and apoptosis induction in oocytes. Consequently, impairments in female reproduction with adverse impacts on oocyte quality would be expected (28).

On the other hand, the growth of embryo might be affected by panic disorder during early pregnancy, and adverse outcomes in the maternal and fetal health would be expected (29).

### SARS-CoV-2 and obesity in female reproduction

Worldwide, people are gaining extra weight during pandemic due to lockdown and limited physical activity, leading to increased obesity rate. The detrimental adverse effect of obesity on fertility and pregnancy has long been detected. Obesity results in hyperinsulinemia and impairment in hypothalamic-pituitary-gonadal (HPG) axis affects ovaries and endometrium. Eventually, obesity results in decline in pregnancy rate, rise in miscarriage and pregnancy complications as well as reduction in rate of still birth (30). Also, obesity is associated with increased risk of poly cystic ovary syndrome (PCOS) which causes anovulation and follicular atresia through ROS (31). Among pregnant women who were hospitalized, obesity was observed in more than a third of them (32). This could give rise to many complications including hypertension, preeclampsia and gestational diabetes in mother. In neonate, heart and neural defects, preterm birth and stillbirth are great risks (33).

### SARS-CoV-2 infection and adverse outcomes in pregnancy

Mixed data regarding effects of SARS-CoV-2 on health of mother and infant/neonate exist, including serious effect on delivery, delivery outcome and vertical transmission.

### Miscarriage and preterm delivery

In contribution to health of infant and neonates, miscarriage appears to not be a concern in infected patients as no significant risk was observed in this population (34, 35). Also, maternal infection may have no effect on infant growth (35-37). Despite this, in case of preterm delivery, some studies indicated higher risk in symptomatic mothers comparing to non-symptomatic/non-infected mothers (32, 34, 38) while others suggested no correlation (35, 37). Yet, based on the fact that the studies supporting higher pre-term delivery in symptomatic patients have a much higher sample size, we author believe SARS-CoV-2 infection increases the risk of pre-term delivery.

In contribution to maternal health, the adverse effects of SARS-CoV-2 before, during and after delivery has been demonstrated in literature. These effects include admission to intensive care unit (ICU), undergoing cesarean and operative vaginal birth and post-partum hemorrhage mainly observed in symptomatic patients along with many other complications (32, 37, 39).

The third trimester of pregnancy was the focal point of most studies on SARS-CoV-2 (34, 35, 40). The complication rate in first and second trimester mothers were similar to non-infected ones (34).

### Vertical transmission of SARS-CoV-2

The vertical transmission could happen via three major routes: i. Placental blood during the course of pregnancy, ii. The birth canal in the course of labor, and iii. During the breastfeeding (41).

Though no sufficient data exist to drive a firm conclusion regarding vertical transmission, based on recent data, the vertical transmission can be deemed to be rare as many studies discussed its possibility (34, 35, 42).

In spite of controversial data, the presence of SARSCoV-2 in placenta has yet to be determined based on further studies (35, 43-45); Though the vertical transmission through placenta has been ruled out based on the observations of Flannery et al. (42) that confirmed the cord blood to contain immunoglobulin G (IgG) without detection of IgM or IgA. The results were verified by other authors (34). Some studies even took a step further to introduce the placenta as a barrier against infection of infants (35, 46). Considering breastfeeding as a vertical transmission mechanism, the same fact applies here (47).

To emphasize on the term “rare”, it is valuable to mention that a few number of cases have been reported “intrauterine transmission”, (48, 49) “placental transmission”, (50, 51) and vertical transmission without mechanistic explanation (52, 53).

### Maternal infection and autism disorder

It is noteworthy to mention that women who had an infection during the second trimester of pregnancy accompanied by a fever are more likely to have children with autism disorder (54). Another study showed that higher levels of IFN-γ, IL-4, and IL-5 were significantly associated with increased risk of autism disorder (55). Thus, it appears that increase in cytokines, particularly IL-6 and IFN-γ during pregnancy may increase the risk of autism disorder.

### Effects of SARS-CoV-2 vaccination on maternal health and pregnancy outcomes

The only data available regarding effects of vaccination on outcome of pregnancy, are from population received Pfizer-BioNTech and Moderna messenger ribonucleic acid (mRNA) based vaccines. More than 28,000 women received these types of vaccines during pregnancy. The reactions one day after vaccination was similar in pregnant and non-pregnant women. Of this population, pregnancy outcome in 827 who completed pregnancy was assessed. One-hundred four (12.6%) had spontaneous abortion which 96 (93.2%) occurred before 13 weeks of gestational age. Out of 712 live births, 700 (98.3%) were vaccinated during the third trimester. After spontaneous abortion, pre-term death was the second most common adverse effect with 9.4% incidence (56).

## Conclusion

The expression of ACE2 and TMPRSS2 in female reproductive system during menstrual cycle and pregnancy (in all three trimesters) has been proven; yet, the mentioned fact does not necessarily mean that infection with SARS-CoV-2 leads to direct effect on female fertility. We believe that the direct effects of SARS-CoV-2 infection are mainly on maternal health before, during and after delivery period causing increased risk of admitting to ICU, caesarian and post-partum hemorrhage among many other complications. Except the risk of pre-term delivery in symptomatic mothers, no other significant risk is threatening the health of infant/ neonate. If any risk exists, it is considered to be rare. Furthermore, vertical transmission from mother to infant/ neonate is rare indicating that adverse effects of SARSCoV-2 on health of infant/neonate is not the consequence of infection in them, rather the consequence of infection in mother and maternal clinical complications.

Nonetheless, the effects of SARS-CoV-2 on female fertility are mainly indirect. The indirect effects are regulated through specific mechanisms, i.e., cytokine storm, psychological disorder and obesity. These mechanisms may lead to increase the risk of pregnancy complications and eventually female infertility.

Safety of SARS-CoV-2 mRNA-based vaccines in pregnant women are not completely verified as pre-term delivery was reported, – although the rate was similar to before pandemic.
